# Topical Colchicine Gel versus Diclofenac Sodium Gel for the Treatment of Actinic Keratoses: A Randomized, Double-Blind Study

**DOI:** 10.1155/2016/5918393

**Published:** 2016-09-05

**Authors:** Gita Faghihi, Azam Elahipoor, Fariba Iraji, Shadi Behfar, Bahareh Abtahi-Naeini

**Affiliations:** ^1^Skin Diseases and Leishmaniasis Research Center, Isfahan University of Medical Sciences, Isfahan, Iran; ^2^Department of Dermatology, Qom University of Medical Sciences, Qom, Iran; ^3^Department of Dermatology, School of Medicine, Rafsanjan University of Medical Sciences, Rafsanjan, Iran; ^4^Cancer Research Center, Semnan University of Medical Sciences, Semnan, Iran

## Abstract

*Introduction*. Actinic keratoses (AKs), a premalignant skin lesion, are a common lesion in fair skin. Although destructive treatment remains the gold standard for AKs, medical therapies may be preferable due to the comfort and reliability .This study aims to compare the effects of topical 1% colchicine gel and 3% diclofenac sodium gel in AKs.* Materials and Methods*. In this randomized double-blind study, 70 lesions were selected. Patients were randomized before receiving either 1% colchicine gel or 3% diclofenac sodium cream twice a day for 6 weeks. Patients were evaluated in terms of their lesion size, treatment complications, and recurrence at 7, 30, 60, and 120 days after treatment.* Results*. The mean of changes in the size was significant in both groups both before and after treatment (<0.001). The mean lesion size before treatment and at 30, 60, and 120 days was not different between the two groups (*p* > 0.05). No case of erythema was seen in the colchicine group, while erythema was seen in 22.9% (eight cases) of patients in the diclofenac sodium group (*p* = 0.005).* Conclusions*. 1% colchicine gel was a safe and effective medication with fewer side effects and lack of recurrence of the lesion.

## 1. Introduction

Actinic keratoses (AKs), also known as premalignant lesions, are a common skin lesion in most communities and are observed in the form of erythematous, scaly lesions in the exposed areas of the skin. It seems that AKs occur where they can develop toward squamous cell carcinoma (SCC), particularly on the head, face, ears, lips, arms, and hands; they are a precancerous lesion [1, 2]. The common symptoms of AKs include painless brown or red scaly macule on sun-exposed areas [1]. Prolonged exposure to sun rays, resulting from outdoor working environments (such as those working in the agricultural sector or engaged in regular outdoor sporting activities), in particular those who have fair skin and are subjected to sun exposure, and compromised immune system by disease or drug have been identified as risk factors affecting the disease, while, so far, the actual etiology of the disease is unknown [3]. AKs will disappear by treatment but new lesions may appear again (particularly at the edges of the treated area) [3]. The possible complication of this disease is the possibility of SCC formation [4].

Surgical or invasive procedures represent the main approach for the treatment of AKs, but noninvasive, tissue-sparing, and topical self-administered treatments may be a highly desirable alternative in both aged and unhealthy patients (who may be poor surgical candidates) as well as for lesions located on cosmetically sensitive areas [5, 6]. While destructive methods of treatment of actinic keratosis remain the gold standard for the eradication of visible and palpable AKs, medical therapies may be able to accomplish this goal with more comfort and reliability for the patient [7].

In the management of multiple AKs, topical therapies include 5% fluorouracil (5-FU), 5% imiquimod (IQ), and 3% diclofenac sodium (DS) gels, which should be preferred over more destructive treatments including surgery, cryotherapy, and curettage surgery and/or invasive treatments [8, 9]. Topical therapy allows the treatment of both visible and subclinical lesions [8]. These treatments showed similar efficacies with different adverse events and cosmetic outcomes. Consequently, it can be seen that guidelines are difficult to construct [8].

Newer topical medications, such as colchicine, ingenol mebutate, and retinoids, are used, but no comparative study has yet been conducted on these drugs with more popular drugs such as DS [10, 11]. Therefore, this study aims to compare the efficacy and safety of topical 1% colchicine gel versus 3% DS gel in the treatment when treating AKs.

## 2. Materials and Methods

### 2.1. Participants

This randomized double-blind study was conducted in Al-Zahra and Noor University Hospitals, Isfahan University of Medical Sciences, Isfahan, Iran, from 2013 to 2014. The protocol of study was approved by the Institutional Review Board of Isfahan University of Medical Sciences and carried out in agreement with the Declaration of Helsinki and its subsequent revisions. After complete explanation of the study details, written informed consent was obtained from eligible patients. This trial was registered at the Iranian Registry of Clinical Trials (IRCT registration number: IRCT2015040721645N1, http://www.irct.ir/searchresult.php?keyword=&id=21645&number=1&prt=8369&total=10&m=1).

The diagnosis of AK was confirmed by two blinded dermatologists before participants were entered into the study. All AKs were located on the face and/or back of the hands and/or scalp in the subjects who were over 18 years of age. The study excluded pregnant or lactating women, patients taking investigational medication, and patients who had received treatment for their lesions within the 8 weeks preceding the study. In addition, patients who had other skin diseases in the area that was to be treated and known sensitivity to any component of the medications under investigation and patients who failed to follow up for various reasons were excluded.

### 2.2. Study Design

Patients underwent a standard clinical assessment and necessary laboratory evaluation 1 week after the onset of the treatment (so that all possible drug complications could be monitored). Overall, 70 patients were selected. Patients were randomized to receive 1% colchicine gel or 3% diclofenac sodium cream in a 1 : 1 ratio using a computer-generated code. Patients underwent a 6-week treatment with one of the two medications. Dermatologist and patients were not informed on the type of treatment and subjects received either treatment A or treatment B by chance. The randomization and allocation process was undertaken by a pharmacist at Al-Zahra Hospital. All patients were instructed to avoid direct sunlight exposure and to use sunscreen. The duration of treatment was twice daily for 6 weeks for both groups.

#### 2.2.1. Medication Preparation

To prepare 1% colchicine gel, the pure colchicine powder (Modava Pharmaceutical Company, Iran) was readied and after being dissolved in water reached the desired volume and percentage on the base of hydroxypropyl methyl cellulose [12].

To prepare the 3% diclofenac cream, the pure powder (Modava Pharmaceutical Company, Iran) was dissolved in water and hydroxypropyl methyl cellulose and 2.5% hyaluronic acid was added to the gel to increase the drug's influence [13].

#### 2.2.2. Outcome Assessment

Two dermatologists conducted a blind evaluation of patients 1 week and 30 and 60 days after the end of treatment and recorded new photographic images (under the same conditions of light and distance in which the first ones were taken).

These dermatologists also conducted a blind evaluation of before and after photographs at the beginning and the end of the treatment. The rate of recovery was considered as complete recovery (complete disappearance of erythema and desquamation) and partial recovery (reduction of erythema, desquamation, and lesion diameter by the scale ruler for dermatology).

Side effects of treatment were systematically recorded throughout the study and were assessed with the use of a checklist, which included pruritus, burning, erythema, and gastrointestinal complication on days 7, 30, 60, and 120.

### 2.3. Statistical Analysis

Statistical analysis was performed using SPSS version 22.0 for Windows; results were presented as mean ± SD. To compare the demographic data and frequency of side effects between the protocols, *t*-test, Fisher's exact test, and chi-square test were performed. Differences were considered significant if *p* ≤ 0.05.

## 3. Results

**Figure 1:**
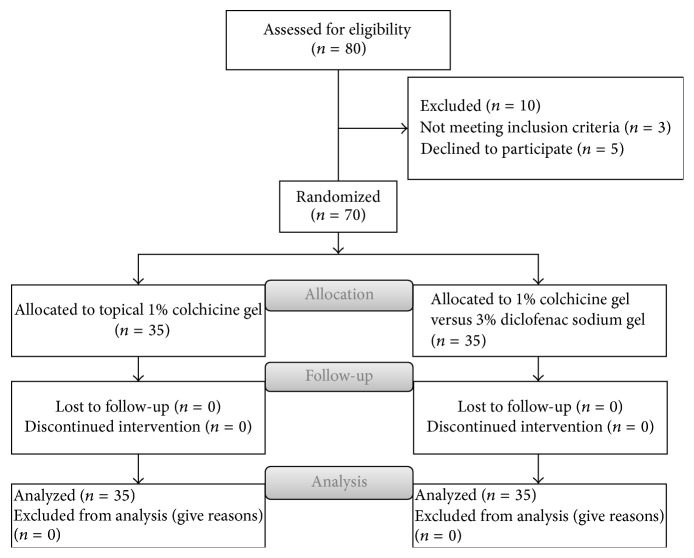
CONSORT flow diagram: topical 1% colchicine gel versus 3% diclofenac sodium gel for the treatment of actinic keratosis.

No significant difference was found between those patients that had been randomly assigned to each group with regard to the basic demographic data including age, gender, and location of lesions. The distributions of age and sex and lesion site are given in Table 1. Also CONSORT flow diagram of the study is given in Figure 1.

The mean of the changes in the size of lesion was significant in both groups both before and after treatment (<0.001).

The mean (±SD) of size of lesions was shown at the start of the treatment and one and two months after the treatment (Table 2).

According to *t*-test, mean (±SD) of surface of lesions had no significant difference between the two groups before treatment (*p* = 0.84) (Table 2).

One month after the treatment, the size of surface of lesions in both groups was reduced to 0.45 ± 0.39 cm^2^ in the group treated by colchicine and 0.39 ± 0.21 cm^2^ in the group treated by diclofenac. According to *t*-test, no significant difference was observed between two groups (*p* = 0.42) (Table 2).

Two months after treatment, the size of surface of lesions in both groups was reduced to 0.23 ± 0.11 cm^2^ in the group treated by colchicine and 0.21 ± 0.11 cm^2^ in the group treated by diclofenac. According to the previously mentioned test, there was no difference between the two groups (*p* = 0.42) (Table 2).

**Figure 2:**
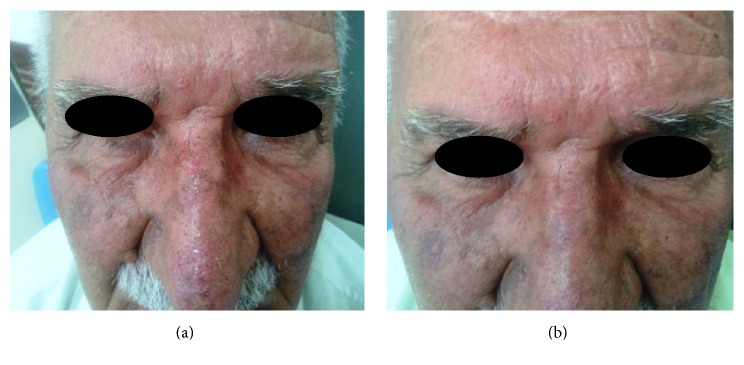
Large AKs on the nose of a participant in the colchicine group (a) at baseline and (b) at the end of the study (8 weeks of treatment).

The clinical efficacy of topical colchicine gel in a representative patient at baseline and at the end of follow-up can be seen in Figure 2.

**Figure 3:**
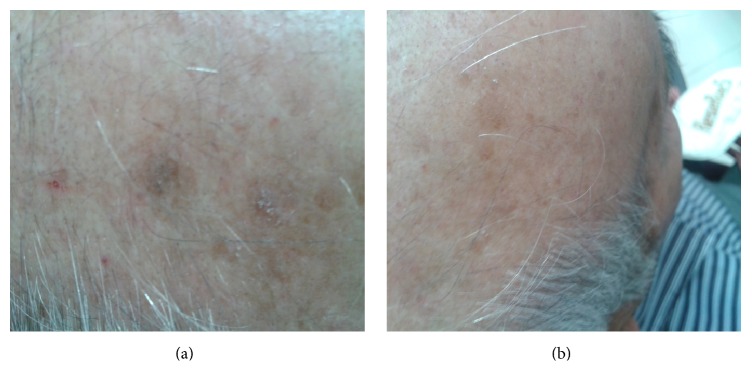
AKs on the scalp of a participant in the diclofenac group (a) at baseline and (b) at the end of the study (8 weeks of treatment).

Although the surface of the lesions was reduced in both groups at 30 and 60 days after treatment, there was no significant difference between the two groups at 30 and 60 days following treatment (Figure 3).

**Figure 4:**
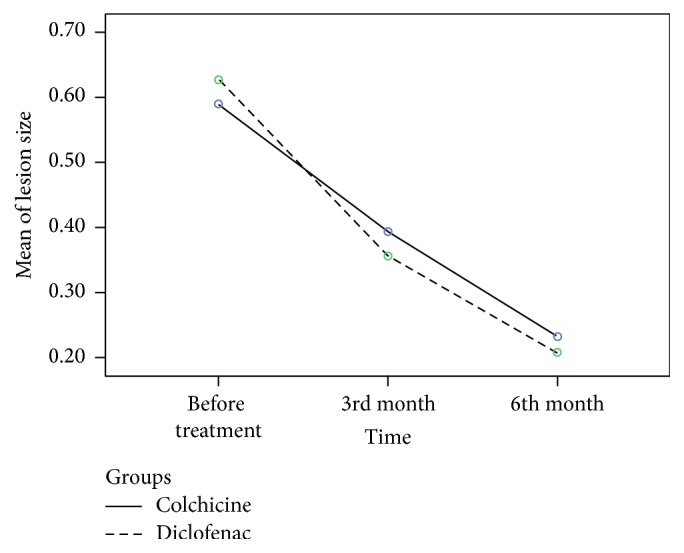
Mean of size of lesions: before and after the treatment. Covariates appearing in the model are evaluated at the following values: sex = 1.2128, age = 63.9362, and place = 1.1702.

The two groups had no significant difference in terms of distribution of age, sex, and site of lesion (>0.05) (Figure 4).

Table 3 shows the percentage of frequency of drug complications as shown in both groups. Overall, 15 cases in colchicine group and 16 cases of the diclofenac sodium group suffered complications as a result of their treatment (42.9% versus 45.7%). According to Fisher's exact test, the complications were the same for both groups (*p* = 0.99).

No case of erythema was seen in colchicine group, while erythema was seen in 22.9% (*n* = 8) of patients in the diclofenac sodium group. This difference was significant (*p* = 0.005). No patient in either group chose to stop their treatment as a result of side effects.

Four months following the end of treatment, the lesions recurred in 2 (5.7%) lesions of the group treated with diclofenac, while no case of recurrence was seen in the group treated by colchicine. According to Fisher's exact test, there was no significant difference in the incidence of recurrence between two groups (*p* = 0.49).

## 4. Discussion

In our studies, colchicine gel was shown to be effective in treating AKs with a 1% concentration gel being applied twice daily for 8 weeks to the face, scalp, trunk, or extremities. Treatment with colchicine and diclofenac led to a significant improvement in the lesions; although a considerable percentage of patients suffered from the complications of treatment, the complications were both mild and tolerable. Colchicine had the capacity to interrupt mitosis and linkage to dimers of tubulin [14]. Such microtubular toxicity results in the cessation of mitosis in metaphase and interference in cellular mobility [15]. This mechanism can explain the clinical effect of colchicine on the treatment of AKs.

In the study by Grimaître et al., the application of a 1% colchicine gel for AKs in double-blind placebo-controlled trials was evaluated. The result of their study showed no recurrence after two months of follow-up. Burning and itching only occurred in patients in the colchicine group two or three days after application, with an inflammatory reaction being seen on those areas where the gel had been applied [12].

Within this study that used colchicine gel, no irritation or erythema was seen in the study participants, and there was no recurrence of lesion up to four months after treatment.

Akar et al. (2001) evaluated the efficacy of different concentrations of topical colchicine applied to AKs. Eight cases were treated with 1% topical colchicine and eight cases with 0.5% topical colchicine. Akar et al.'s (2001) results showed that topical colchicine is an effective and safe alternative agent for the treatment of AKs. Cream containing 0.5% colchicine is equally effective as 1% colchicine cream when treating AKs [16].

A meta-analysis of three studies for treatment of AKs with diclofenac 3% gel in 2.5% hyaluronic acid with a total of 364 patients revealed complete remission in 39.1% of patients [17].

Systemic toxicity with colchicine is a concern, and it is known that colchicine and its analogs interfere with microtubule growth within nerve cells, ciliated cells, leukocytes, and sperm [18].

Colchicine forms high-affinity complexes with tubulin and inhibits this protein's polymerization. Microtubule assembly and elongation are, therefore, disrupted, limiting the chemotactic and phagocytic activity of polymorphonuclear lymphocytes [19, 20].

Although the patients in this study only received topical colchicine, they were monitored closely for clinical signs of systemic toxicity such as hematologic side effects, including pancytopenia.

None of our patients demonstrated any systemic adverse events.

Our experiences with colchicine suggest that this effective treatment modality is a useful option for patients with AKs. There appears to be a low risk of systemic or local toxicity with this regimen. The data suggest that a more randomized, blinded, and controlled clinical trial using a larger sample size was needed in order to establish the true efficacy of colchicine.

Our study had some limitations, including small sample size and short duration of follow-up. Consequently, further comparative studies for clinical evaluation are recommended.

## 5. Conclusion

The results of the study show the use of topical 1% colchicine gel and 3% diclofenac sodium gel for the treatment of AKs to be both safe and effective treatment for AKs. The lack of long-term erythema and recurrence of the lesion is encouraging for use of topical colchicine gel.

## Figures and Tables

**Table 1:** Distribution of age and sex and location of the lesion in two groups separately.

Variables		Groups		
		Colchicine gel	Diclofenac gel	*p* value
Mean (±SD) of age		63.7 ± 9.2	62.3 ± 8.4	0.48

Sex *N* (%)	Male	26 (74.3)	30 (85.7)	0.23
	Female	9 (25.7)	5 (14.3)	

Location *N* (%)	Face	27 (77.1)	29 (82.9)	0.55
	Scalp	8 (22.9)	6 (17.1)	

**Table 2:** Mean (±SD) of surface of lesion: before and after the treatment.

Time	Groups		
	Colchicine gel	Diclofenac gel	*p* value
Before treatment	0.65 ± 0.37	0.65 ± 0.21	0.84
30 days later	0.39 ± 0.21	0.45 ± 0.39	0.42
60 days later	0.21 ± 0.11	0.23 ± 0.11	0.62
*p* value	<0.001	<0.001	—

**Table 3:** Frequency of the incidence of complications during treatment in both groups.

Side effects (*n*)		Colchicine (*n*/%)	Diclofenac (*n*/%)	*p* value
Pruritus	Yes (15)	7 (20)	8 (22.9)	0.99
	No (55)	28 (80)	27 (77.1)	

Burning	Yes (17)	10 (28.5)	7 (20)	0.57
	No (53)	25 (71.5)	28 (80)	

Erythema	Yes (8)	0 (0)	8 (22.9)	0.005
	No (62)	35 (100)	27 (77.1)	

Infection	Yes (0)	0 (0)	0 (0)	
	No (70)	35 (100)	35 (100)	

Gastrointestinal complication	Yes (31)	15 (42.9)	16 (45.7)	0.99
	No (39)	20 (57.1)	19 (54.3)	
