# Corrigendum to “Topical Colchicine Gel versus Diclofenac Sodium Gel for the Treatment of Actinic Keratoses: A Randomized, Double-Blind Study”

**DOI:** 10.1155/2017/7192180

**Published:** 2017-12-28

**Authors:** Gita Faghihi, Azam Elahipoor, Fariba Iraji, Shadi Behfar, Bahareh Abtahi-Naeini, Fatemeh Mokhtari

**Affiliations:** ^1^Skin Diseases and Leishmaniasis Research Center, Isfahan University of Medical Sciences, Isfahan, Iran; ^2^Department of Dermatology, Qom University of Medical Sciences, Qom, Iran; ^3^Department of Dermatology, School of Medicine, Rafsanjan University of Medical Sciences, Rafsanjan, Iran; ^4^Cancer Research Center, Semnan University of Medical Sciences, Semnan, Iran; ^5^Dermatology Department, School of Medicine, Isfahan University of Medical Sciences, Isfahan, Iran

In the article titled “Topical Colchicine Gel versus Diclofenac Sodium Gel for the Treatment of Actinic Keratoses: A Randomized, Double-Blind Study” [1], Dr. Fatemeh Mokhtari was missing from the authors' list. The corrected authors' list is shown above.
